# HIV vaccine acceptability among high-risk drug users in Appalachia: a cross-sectional study

**DOI:** 10.1186/1471-2458-14-537

**Published:** 2014-05-30

**Authors:** April M Young, Ralph J DiClemente, Daniel S Halgin, Claire E Sterk, Jennifer R Havens

**Affiliations:** 1 Department of Epidemiology, University of Kentucky College of Public Health, 111 Washington Avenue, Lexington, Kentucky 40536, USA; 2 Center on Drug and Alcohol Research, University of Kentucky, 333 Waller Avenue, Lexington, KY 40504, USA; 3 Department of Behavioral Sciences and Health Education, Emory University, 1518 Clifton Road, Atlanta, GA 30322, USA; 4 LINKS Center for Social Network Analysis, Gatton College of Business and Economics, University of Kentucky, 550 S Limestone St, Lexington, KY 40526, USA

**Keywords:** HIV, Vaccination, AIDS vaccines, HIV vaccines, Attitude, Drug users, Psychological theory, Rural health

## Abstract

**Background:**

A vaccine could substantially impact the HIV epidemic, but inadequate uptake is a serious concern. Unfortunately, people who use drugs, particularly those residing in rural communities, have been underrepresented in previous research on HIV vaccine acceptability. This study examined HIV vaccine acceptability among high-risk drug users in a rural community in the United States.

**Methods:**

Interviewer-administered questionnaires included questions about risk behavior and attitudes toward HIV vaccination from 433 HIV-negative drug users (76% with history of injection) enrolled in a cohort study in Central Appalachia. HIV vaccine acceptability was measured on a 4-point Likert scale. Generalized linear mixed models were used to determine correlates to self-report of being “very likely” to receive a 90% effective HIV vaccine (i.e. “maximum vaccine acceptability”, or MVA). Adjusted odds ratios (AORs) and corresponding 95% confidence intervals (CIs) are reported.

**Results:**

Most (91%) reported that they would accept a preventive HIV vaccine, but concerns about cost, dosing, transportation constraints, vaccine-induced seropositivity, and confidentiality were expressed. Cash incentives, oral-administration, and peer/partner encouragement were anticipated facilitators of uptake. In multivariate analysis, men were significantly less likely to report MVA (AOR: 0.33, CI: 0.21 – 0.52). MVA was more common among participants who believed that they were susceptible to HIV (AOR: 2.31, CI: 1.28 – 4.07), that an HIV vaccine would benefit them (AOR: 2.80, CI: 1.70 – 4.64), and who had positive experiential attitudes toward HIV vaccination (AOR: 1.85, CI: 1.08 – 3.17). MVA was also more common among participants who believed that others would encourage them to get vaccinated and anticipated that their behavior would be influenced by others' encouragement (AOR: 1.81, 95% 1.09 – 3.01).

**Conclusions:**

To our knowledge, this study was among the first to explore and provide evidence for feasibility of HIV vaccination in a rural, high-risk population in the United States. This study provides preliminary evidence that gender-specific targeting in vaccine promotion may be necessary to promoting vaccine uptake in this setting, particularly among men. The data also underscore the importance of addressing perceived risks and benefits, social norms, and logistical constraints in efforts to achieve widespread vaccine coverage in this high-risk population.

## Background

Since 1987, the scientific community has been in pursuit of an effective HIV vaccine [1]. In response to the possibility that an HIV vaccine is on the horizon, researchers have mobilized to examine the feasibility of disseminating the vaccine. In high-risk populations around the world, numerous studies on HIV vaccine acceptability have been conducted, but people who use drugs have been underrepresented. Recent research investigating attitudes toward compulsory HIV vaccination among high-risk individuals in Los Angeles found that that people who inject drugs (PWID) were significantly less likely to endorse universal vaccination or vaccination of all children or adults compared to their non-injecting counterparts [2]. The authors point out that the strong opposition to compulsory vaccination policies among PWID may be indicate future challenges in HIV vaccine acceptance and dissemination [2], thus reinforcing the need for additional research. Of the 15 quantitative studies from the US included in a recent review [3], only three included drug users [4-6]; none of which reported results stratified by drug use. Qualitative studies on HIV vaccine acceptability are equally limited, as nearly all of those involving people who use drugs have been conducted in one setting (i.e. Los Angeles) [6-11]. There are no studies to date evaluating HIV vaccine acceptability in a high-risk, rural drug-using population in the US. National surveillance data indicate that while the prevalence of AIDS has gradually declined in most urban areas since the mid 1980's, the number of cases continues to slowly increase in many rural communities, particularly in the South [12,13]. Given the historically low prevalence of HIV in rural areas and the common misconception that HIV is an “urban problem”, many rural communities are unequipped to deal with the social, economic, and healthcare burden posed by an increase in HIV.

Central Appalachia, which encompasses some of the most economically distressed counties in the US [14], would face many of the challenges posed by an increase in HIV and AIDS. The Appalachian region is characterized by marked health disparities [15], an under-resourced health infrastructure [15], and prevalent misuse of prescription drugs [16-18]. While HIV prevalence is currently low in this population [19], recent evidence from Eastern Kentucky, in Central Appalachia, suggested that many nonmedical prescription drug users were infected with hepatitis C [20], had engaged in injection drug use (IDU) [21] and frequent unprotected sex [22], and were embedded in a highly cohesive and centralized risk network that could facilitate HIV transmission [23]. Given these risk factors, stigma surrounding HIV [24], and myriad cultural and socioeconomic complexities, Central Appalachia is a setting in which greater knowledge of potential barriers and facilitators to HIV vaccine acceptability will be essential in achieving adequate coverage. The purpose of this study was to examine demographic, behavioral, and psychosocial correlates to HIV vaccine acceptability among a sample of HIV negative, high-risk drug users in Central Appalachia.

## Methods

### Sample

The data used for this analysis were collected during the 24-month assessment of the longitudinal Social Networks among Appalachian People (SNAP) study. Recruitment and assessment are described in detail elsewhere [20,23,25]. To be eligible, participants were required to be age 18 or older, reside in Appalachian Kentucky, and to have used prescription opioids, heroin, crack/cocaine, or methamphetamine to get high in the prior 30 days. Participants (n = 503) were recruited from rural Appalachian Kentucky using respondent-driven sampling and data were collected using questionnaires administered by trained community-based staff. Participants completed follow-up interviews and HIV testing at 6-month intervals. The 24-month interview was completed by 435 participants between March 2012 and May 2013.

### Measures

Following their 24-month interview, participants (n = 433) were invited to complete an interviewer-administered questionnaire on their attitudes toward HIV vaccination (two who were interviewed in jail were not invited due to time-constraints). All invited participants consented and were compensated $35 for participation. Before the questionnaire, interviewers read a script reminding them that HIV can be transmitted through sharing drug equipment and having unprotected sex, that HIV is the cause of AIDS, and that there is currently no cure. The script informed participants that the vaccine referred to throughout the questionnaire would not cure HIV, but would prevent acquisition. A 90% efficacy level was specified for the questions presented in these analyses. The University of Kentucky's Institutional Review Board approved the protocol.

#### HIV vaccine acceptability

HIV vaccine acceptability was assessed with: “Imagine that an affordable HIV vaccine was approved and made available to you in the next 12 months. This vaccine would prevent you from getting HIV almost all of the time (90% effective). How likely would you be to get this vaccine?” followed by a 4-point Likert scale ranging from ‘very unlikely’ to ‘very likely’. Due to skewness, the responses were dichotomized for analysis (0 = Very unlikely/Unlikely/Likely; 1 = Very likely). Given the debatable association between these intentions and actual behavior [26], this conservative dichotomization may provide a better indication of future uptake. Hereafter, those who were 'very likely' to accept the vaccine are referred to as reporting “maximum vaccine acceptability” (MVA).

#### Vaccine characteristics

Participants were also asked about vaccine characteristics identified in previous research as factors relevant to acceptability [3,5,6,27,28]. Items assessed willingness to pay (continuous), minimum acceptable vaccine efficacy (ordinal in increments of 10%), and whether cash incentives, dosing (multiple vs. single), administration (oral vs. injected), and/or vaccine-induced positive results on future HIV tests would affect vaccine acceptability.

#### Demographic, behavioral, and psychosocial measures

Basic demographic and behavioral data were also collected (listed in Table 1). The psychosocial measures were based on a modified version [29] of the Integrative Model (IM) [30], which posits that behavior is directly affected by intention. Intention is influenced by attitudes, perceived norms, and personal agency, which are in turn influenced by background factors (e.g., demographic, behavioral, and other contextual characteristics). Table 2 describes the items and coding scheme used to assess the following: attitudes (instrumental and experiential), subjective norms (descriptive and injunctive), and personal agency (perceived behavioral control and self-efficacy). Due to skew in the response distribution of the four-point Likert-scale and semantic differential scale measures, items were dichotomized at the mid-point of the ‘forced choice’ style response options such that a value of 1 indicated a positive response and a value of 0 indicated a negative response. *Experiential* and *instrumental attitudes* refers to emotional and cognitive responses, respectively, to performing a behavior [31]. Experiential attitudes were examined with semantic differential scale items used in a similar study [32]. The *instrumental attitude* measures were adapted from the Health Belief Model [33]; these include perceived severity of and susceptibility to HIV, and perceived benefits of and barriers to HIV vaccination.

**Table 1:** Demographic and behavioral characteristics of the sample (n = 433)

**Characteristic**	**N (%)**
*Demographic*	
Male	239 (55.2)
Age – median (IQR)	34 (29 – 41)
White	407 (94.0)
High school graduate	251 (58.0)
Married	111 (25.6)
Unemployed	169 (39.0)
Income in past 30 days^a^ – median (IQR)	$698 (200 – 1100)
Uninsured	285 (65.8)
*Drug use in past 6 months*	
Nonmedical use of prescription drugs^b^	368 (95.0)
Cocaine	51 (11.8)
Methamphetamine	35 (8.1)
Heroin	23 (5.3)
Crack	14 (3.2)
*IDU-related behaviors* (past 6 months)	
Injected drugs at least once	146 (33.7)
Injected with unclean needle	33 (7.6)
Gave/loaned/sold an unclean needle	16 (3.7)
Shared injection equipment^c^	55 (12.7)
*Sexual behavior* (past 6 months)	
Number of sex partners	
Zero	76 (17.6)
One partner	254 (58.7)
Two partners	56 (12.9)
Three or more partners	47 (10.9)
Unprotected sex with at least one partner	308 (71.1)
Unprotected sex with PWID	85 (19.6)

IQR: interquartile range; PWID: person who injects drugs; IDU: injection drug use.

^a^Includes income from employment, unemployment compensation, welfare, pension/social security, child support, friends/family, and illegal activities.

^b^Includes nonmedical use of methadone, OxyContin, oxycodone, buprenorphine, Roxicodone, hydrocodone, other opiates (e.g., Neurontin, Ultram, morphine, Demerol, Opana, Embeda, Avinza), and benzodiazepines.

^c^ Cookers, cottons, and/or rinse water.

**Table 2:** Psychosocial attitudes about HIV vaccination in sample of rural drug users

**Construct**	**Measure**	**Dichotomized response**	**N (%)**
*Attitudes (n = 433)*			
Severity	In your opinion, how serious would it be if you were infected with HIV?^b^	Very/Extremely Serious	427 (98.6)
Susceptibility	If you did not get a vaccine, how likely do you think you would be to get HIV in your lifetime?^b^	Likely/Very Likely	100 (23.1)
Benefits	In your opinion, how much would an HIV vaccine benefit you?^c^	Some/A lot	313 (72.3)
Barriers	What factors would make it difficult for you to receive the HIV vaccine? [see Figure 1]	[Endorsed at least one barrier]	344 (79.4)
Experiential	[3-items] For me, getting an HIV vaccine would be… ["stressful - relaxing", “frightening – comforting”, “irresponsible – responsible”]^a^	[Positive rating on all three items]	343 (79.2)
*Subjective norms (n = 432)*			
Descriptive norm		*Affirmative response on the following two items*	195 (45.1)
Normative belief	If an HIV vaccine became available, most people important to me would get it.^d^	Agree/Strongly Agree	358 (82.9)
Motivation to comply	If most people got the HIV vaccine, would you be [More likely to get it/Less likely to get it/Would not affect my decision]^e^	More likely to get it	218 (50.5)
Injunctive norm		*Affirmative response on the following two items*	251 (58.1)
Normative belief	Most people important to me would be supportive of me getting the HIV vaccine.^d^	Agree/Strongly Agree	408 (94.4)
Motivation to comply	If most people encouraged you to get the HIV vaccine, would you be [More likely to get it/Less likely to get it/Would not affect my decision]^e^	More likely to get it	256 (59.3)
*Personal agency*			
Behavioral control	How much personal control do you feel that you would have over getting the HIV vaccine?^f^	A lot/Complete control	276 (63.7)
Self-efficacy	How sure are you that you could get the HIV vaccine if …^g^	*Affirmative response each of the following 3 items*	83 (19.2)
	…you had to pay for it out of pocket?	Very/Extremely Sure	105 (24.2)
	…you had to travel out of town to get it?	Very/Extremely Sure	184 (42.5)
	…your friends/partners did not want you to get it?	Very/Extremely Sure	266 (61.4)

^a^Measured on 4-point semantic differential scales; dichotomized where 1 = rating of three or four on all items, 0 = rating of one or two on at least one item.

^b^Measured on 4-point scale dichotomized where: 0 = Very unlikely/Unlikely, 1 = Likely/Very likely.

^c^Measured on 4-point scale dichotomized where: 0 = Not at all/Little, 1 = Some/a lot.

^d^Measured on 4-point scale dichotomized where 0 = Strongly disagree/Disagree, 1 = Agree/Strongly agree.

^e^Dichotomized where 0 = Less likely to get it/Would not affect my decision, 1 = More likely to get it.

^f^Measured on 4-point scale dichotomized where 0 = No control/Some control, 1 = A lot of control/Complete control.

^g^Each measured on 4-point scales dichotomized where 0 = Not sure at all/Somewhat sure, 1 = Very sure/Extremely sure. Total measure was dichotomized where 1 = rating of one on all items, 0 = rating of zero on at least one item.

*Injunctive norms* are a person's beliefs about and motivation to comply with what others think he/she should do. *Descriptive norms* refer to a person's perceptions about others' behavior and his/her motivation to comply with (i.e. imitate) their actions [34,35]. Descriptive and injunctive norms are each comprised of two sub-constructs: normative beliefs and motivation to comply. Self-efficacy and perceived behavioral control were also examined. *Self-efficacy* is the belief in one's general capabilities to exercise control over his/her behavior [36], while *perceived behavioral control* focuses on one's abilities to perform a behavior in light of various barriers [37].

### Statistical analyses

Given potential autocorrelation among responses, generalized linear mixed models were used. Models were estimated using the PROC GLIMMIX [38] procedure (SAS software, version 9.3) with a random effect for subject and Laplace approximation [39]. To adjust for potential biases presented by respondent-driven sampling [40,41], individualized weights computed in RDSAT 7.1 (Ithaca, NY) [42] were used in all analyses. The weights were based on individual network size and partition analysis on the dependent variable using enhanced data smoothing and 25,000 bootstrap iterations. Odds ratios (ORs), adjusted odds ratios (AORs), and 95% confidence intervals (CIs) were reported. Each demographic and behavioral variable was assessed independently for its association with the outcome, and those reaching significance (p < 0.05) were entered into multivariate analyses. Due to the a priori nature of the IM, all psychosocial variables were entered into multivariate analyses regardless of bivariate significance, as suggested in previous research [43].

## Results

Descriptive demographic and behavioral data are presented in Table 1. Briefly, the median age was 34 years (range: 21–68), 55% were male, and most respondents were White (94%); the latter is reflective of the demographic profile of Central Appalachia [44]. Most (76%) reported a lifetime history of IDU and 34% reported recent IDU (past 6 months). Receptive and distributive needle sharing were uncommon, but 13% had shared other injection paraphernalia. Approximately 24% reported multiple sex partners in the past 6 months and 71% had unprotected sex, including 20% who had done so with PWID.

### Attitudes toward HIV and HIV vaccination

**Figure 1:**
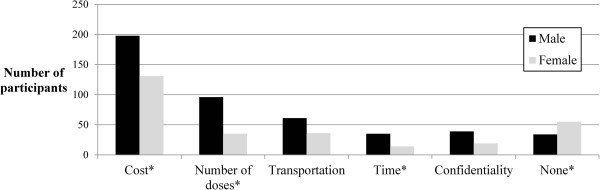
**Anticipated barriers to HIV vaccine acceptability among men and women (n = 433).** An asterisk (*) indicates a statistically significant difference (p < 0.05) between men and women based on chi-square analysis.

Most reported that they would be very likely (59%) or likely (32%) to receive an HIV vaccine. Psychosocial attitudes are shown in Table 2 and anticipated barriers, stratified by gender, are displayed in Figure 1. Of note, men were significantly more likely to report cost, requirement for multiple doses, and time as barriers to vaccine acceptability; women were more likely to report that there were no barriers to vaccine acceptability. Overall, 76% were unsure or only somewhat sure that they could get vaccinated if they had to pay out-of-pocket, travel out of town to get it (58%), or if their friends/partner were unsupportive (39%). Most (83%) reported that most people they knew would accept the vaccine, but only 51% would be more likely to accept the vaccine if most people did so. Similarly, 94% believed that most people would be supportive of their vaccination and 60% would be more likely to be vaccinated if most people encouraged them.

Table 3 describes attitudes toward specific vaccine characteristics. Most reported that requirement for multiple doses would not influence vaccine acceptability; however, 44% reported that they would be more likely to accept an orally-administered vaccine. Most (62%) reported that they would be more likely to get vaccinated if there was a cash incentive to do so; the median incentive amount necessary to motivate vaccination was $50. The majority (56%) reported that the vaccine would need to be 90% effective before they would agree to be vaccinated and 18% reported that the vaccine would need to provide complete protection. Nearly all (93%) were willing to pay for a 90% effective vaccine; the median price participants were willing to pay was $100.

**Table 3:** Characteristics of HIV vaccination that could facilitate or hinder vaccine acceptability

**Characteristic**	**N(%)**
*Price (USD) willingness to pay for a 90*% *effective HIV vaccine*	
$0	30 (6.9)
$1 - $50	126 (29.1)
$51 - $100	132 (30.5)
$101 - $200	57 (13.2)
$201 - $500	51 (11.8)
$501 - $1000	28 (6.5)
Greater than $1000	9 (2.1)
*Efficacy required before participant would accept an HIV vaccine (n = 423)*^*a*^	
Less than 50%	5 (1.2)
50%	37 (8.7)
60%	5 (1.2)
70%	13 (3.1)
80%	52 (12.3)
90%	237 (56.0)
100%	74 (17.5)
*Factors that would make participant less likely to get the vaccine (n = 431)*	
Requirement for multiple doses (versus single dose)	86 (20.0)
Caused future HIV test results to be positive	221 (51.3)
*Factors that would make participant more likely to get the vaccine*	
Cash incentive (n = 431)	269 (62.4)
Amount necessary to motivate vaccination (USD) (n = 269)	
Less than $20	16 (5.9)
$20 - $50	147 (54.6)
$51 - $100	81 (30.1)
$101 - $500	18 (6.7)
$501 - $1000	4 (1.5)
$1001 - $2000	2 (0.7)
$10,000	1 (0.4)
Orally administered rather than injected (n = 430)	190 (44.2)

^a^Missing data include nine participants who reported that they would not accept the vaccine regardless of efficacy.

### Bivariate and multivariate results

Bivariate results are presented in Table 4. Men and older participants were significantly less likely to report MVA. Injection drug use, use of an unclean needle, and unprotected sex with PWID in the past 6 months was positively associated with MVA. Perceived susceptibility to HIV, perceived benefit of the vaccine, positive experiential attitudes, and perceived behavioral control were also positively associated with MVA. Respondents reporting that people important to them would accept an HIV vaccine *and* that they would be more likely to accept the vaccine if others did so were more likely to report MVA. Similarly, those who believed that people would encourage them to receive the vaccine and who reported being more likely to accept the vaccine if others encouraged them were nearly twice as likely to report MVA.

**Table 4:** Bivariate correlates to vaccine acceptability (n = 433)

**Characteristic**	**Vaccine acceptability**		**Bivariate**	
	**Not very likely**^**a **^**(n = 176)**	**Very likely (n = 257)**	**OR (95% CI)**	**p-value**
*Demographic*				
Male	122 (69.3)	117 (45.5)	0.24 (0.12- 0.48)	<0.001^**^
White	163 (92.6)	244 (94.9)	2.16 (0.68 – 6.90)	0.194
Age - mean (SD)	36.3 (9.3)	34.9 (8.1)	0.96 (0.94 – 0.99)	0.018^*^
Income (n = 432) - mean (SD)	$908 (1473)	$913 (1125)	1.00 (1.00 - 1.00)	0.629
High school graduate	93 (52.8)	158 (61.5)	1.63 (0.93 – 2.85)	0.088
Uninsured	114 (64.8)	171 (66.5)	1.32 (0.76 – 2.30)	0.331
Married	41 (23.3)	70 (27.2)	1.58 (0.85 – 2.93)	0.144
*Behavioral* (past 6 months)				
Injected drugs	46 (26.1)	100 (38.9)	2.54 (1.41 – 4.58)	0.002^**^
Injected with unclean needle	9 (5.1)	24 (9.3)	4.53 (1.53 – 13.39)	0.006^**^
Distributed unclean needle^b^	5 (2.8)	11 (4.3)	3.23 (0.72 – 14.38)	0.125
Shared injection equipment^c^	18 (10.2)	37 (14.4)	2.02 (0.90 – 4.55)	0.090
Had multiple sex partners	35 (19.9)	68 (26.5)	1.53 (0.78 – 2.98)	0.213
Had unprotected sex	120 (68.2)	188 (73.2)	1.49 (0.68 – 2.66)	0.180
Unprotected sex with PWID	24 (13.6)	61 (23.7)	3.33 (1.64 – 6.76)	0.001^**^
*Attitudes about HIV*				
Severity of HIV	173 (98.3)	254 (98.8)	1.53 (0.13 – 18.25)	0.738
Susceptibility to HIV	23 (13.1)	77 (30.0)	4.63 (2.17 – 9.90)	<0.001^**^
Benefits of HIV vaccine	103 (58.5)	210 (81.7)	5.85 (2.76 – 12.40)	<0.001^**^
Barriers to HIV vaccination	149 (84.7)	195 (75.9)	0.52 (0.26 – 1.03)	0.060
Experiential attitude	127 (72.2)	216 (84.0)	3.14 (1.60 – 6.16)	0.001^**^
*Subjective norms*				
Descriptive norms	65 (37.1)	130 (50.6)	2.36 (1.34 – 4.18)	0.003^**^
Injunctive norms	85 (48.6)	166 (64.6)	2.67 (1.47 – 11.13)	0.001^**^
*Agency*				
Behavioral control	101 (57.4)	175 (68.1)	1.88 (1.05 – 3.34)	0.032^*^
Self-efficacy	26 (14.8)	57 (22.2)	2.01 (0.98 – 4.13)	0.058

PWID: person who injects drugs; OR: odds ratio; CI: confidence interval; SD: standard deviation.

^*^p < 0.05; ^**^p < 0.01.

^a^Includes responses “very unlikely”, “unlikely”, and “likely”.

^b^Sold, loaned, or gave needle to someone after using it.

^c^Cookers, cottons, and/or rinse water.

Multivariate results are described in Table 5. Controlling for other variables in the model, men were less likely to report MVA (AOR: 0.33, CI: 0.21 - 0.52). Participants who believed they were susceptible to HIV (AOR: 2.31, CI: 1.28 - 4.17), perceived that the vaccine would benefit them (AOR: 2.80, CI: 1.70 - 4.64), and reported positive experiential attitudes (AOR: 1.85, CI: 1.09 - 3.01) were significantly more likely to report MVA. Injunctive norms were also positively associated with vaccine acceptability (AOR: 1.81, CI: 1.09 – 3.01).

**Table 5:** **Multivariate correlates to being “very likely” to receive an HIV vaccine (n = 432)**^**a**^

**Characteristic**	**AOR (95% CI)**	**p-value**
*Demographic*		
Male	0.33 (0.21 - 0.52)	<0.001^**^
Age	1.00 (0.98 – 1.03)	0.872
*Behavioral (past 6 months)*		
Injected drugs	1.25 (0.70– 2.26)	0.453
Injected drugs with unclean needle	0.80 (0.29 – 2.20)	0.659
Bleached injection equipment	1.05 (0.39 – 2.82)	0.925
Unprotected sex with PWID	1.42 (0.72 – 2.80)	0.312
*Attitudes*		
Perceived severity of HIV	0.67 (0.11 – 4.07)	0.664
Perceived susceptibility to HIV^2^	2.31(1.28 – 4.16)	0.006^**^
Perceived benefits	2.80 (1.70 – 4.64)	<0.001^**^
Perceived barriers	0.62 (0.32 - 1.23)	0.175
Experiential attitude	1.85 (1.08 – 3.17)	0.025^*^
*Subjective norms*		
Descriptive norms	1.17 (0.70 – 1.95)	0.552
Injunctive norms	1.81 (1.09 – 3.01)	0.023^*^
*Agency*		
Perceived behavioral control	1.25 (0.77 – 2.01)	0.363
Self-efficacy	1.27 (0.65 – 2.52)	0.485

PWID: person who injects drugs; AOR: adjusted odds ratio; CI: confidence interval.

*p < 0.05; ^**^p < 0.01.

^a^Data on norms were missing for one participant resulting in the inclusion of 432 in the analysis.

## Discussion

In this sample of rural drug users, 91% were likely or very likely to accept a 90% effective, preventive HIV vaccine. This percentage is comparable to that found in other urban and suburban populations in the US [45-47]. Men were significantly less likely to indicate that they were very likely to receive an HIV vaccine, after adjustment for behavioral characteristics and psychosocial constructs. Previous research on the association between gender and HIV vaccine acceptability is mixed, with one study finding that acceptability was higher among women [27] and another finding that it was higher among men [48]. Research conducted among high-risk adults in Los Angeles identified gender differences in concerns and motivations surrounding HIV vaccination (e.g., women were more likely to be influenced by factors related to their intimate relationships and experiences with healthcare providers, while men were more influenced by peer perceptions and risk of vaccine-induced seropositivity), but no significant association between gender and vaccine acceptability [49]. In the present study, differences in vaccination concerns, specifically those related to perceived barriers, may play an important role in the observed gender difference in vaccine acceptability. Men were significantly more likely to report that cost, requirement for multiple doses (vs. a single dose), and time to visit the clinic would be barriers to vaccine acceptability. Post-hoc analysis to investigate these patterns revealed no significant gender differences in unemployment or *total* monthly income; however, men reported significantly more monthly income from employment and women reported significantly more income from partners, peers, family, and child support. Men were also more likely to report being uninsured. Interestingly, there was no gender difference in the amount participants were willing to pay for the vaccine. These patterns may indicate that while men and women have similar total gross incomes and willingness to pay, men have less net income to use for purchasing an HIV vaccine and the insurance coverage to reduce out-of-pocket costs.

Findings regarding the association between gender and barriers posed by dosing and time constraints would seem to indicate that men anticipate experiencing other logistical obstacles to accessing vaccination. Unavailability of time to visit the clinic for one or multiple doses may be related to a number of factors, including employment. Post-hoc analyses revealed no association between gender and past 6-month unemployment or full-time employment, but men were more likely to report part-time, irregular day work. The location, hours, and nature of this work is largely unknown, as are details about additional obligations that could compete with time available to seek vaccination. Although gender differences in psychosocial constructs aside from perceived barriers (e.g., perceived susceptibility to HIV, perceived severity of HIV) were not observed, it is important to note that perceived and/or actual barriers to vaccination are likely only part of the confluence of factors that could contribute to gender differences in HIV vaccine acceptability in this and other settings. In this setting, a “one-size-fits-all” [50] approach to mitigating barriers to HIV vaccination may not be appropriate. Strategies that consider possible gender differences in constraints on HIV vaccine acceptance and, in turn, “meet men and women where they are” psychologically, socially, and geographically should be developed. These approaches may include strategic location of vaccine dissemination sites (e.g., at worksites, clinics, mobile units), varied hours of availability, and payment structures that limit out-of-pocket costs to those with and without insurance.

Given their low income and high rate of unemployment, it is unsurprising that most participants reported that cost would be a barrier to vaccine acceptability. Cost has been identified as an important influence in HIV vaccine acceptability in many [3,27,28], but not all [5,51] previous studies. Interestingly, participants in one study believed that the vaccine should be given at some cost, as free services were often perceived as inferior to those that were purchased [52]. Nearly all participants in the present study were willing to pay for a 90% effective HIV vaccine, but only one-third would be able to afford out-of-pocket costs exceeding $100. Though some research has suggested that cost may not be as strongly associated with acceptability as are other vaccine characteristics [5], in this setting, minimization of out-of-pocket costs would be critical to achieving adequate coverage.

The majority (60%) reported that a modest cash incentive (less than $50) would improve their likelihood of accepting the vaccine. Previous research among PWID demonstrated that monetary incentives can improve compliance with a three-dose hepatitis B vaccine regimen [47]; the cost-effectiveness of a similar approach to HIV vaccination should be considered. Monetary incentives may assist in offsetting logistical costs, such as transportation, which was reported as a barrier to acceptability by a sizable minority of participants. This finding underscores, as has previous research [3], the importance of minimizing logistical constraints on accessing vaccination. However, this must be coupled with interventions which address concerns regarding confidentiality; nearly one in eight were concerned that providers would disclose their vaccination status to others. Participants in several previous studies have reported concern about peers’ negative social reactions to HIV vaccination [7,49,52-55], though less is known about participants’ confidentiality concerns related to healthcare providers administering the vaccine. Participants in a qualitative study in Los Angeles reported concern about being seen at vaccine dissemination sites [7] and one study in Thailand found that PWID were concerned about being seen at vaccine dissemination locations due to fear of legal consequences (e.g., arrest) [55]. In this and other settings, appropriate selection of vaccine dissemination sites as well as intensive training of providers about confidentiality will be critical to ensuring adequate vaccine coverage among high-risk populations.

Findings regarding the importance of perceived social norms may also inform appropriate and effective strategies for HIV vaccine promotion. Descriptive data revealed that nearly 40% were not sure or only somewhat sure that they would be able to get the HIV vaccine if a friend/partner was unsupportive. Several previous studies have indicated that peer support and positive social norms will be important for facilitating vaccine acceptability. Participants in previous studies have reported fear of negative reactions by family members [7,49] and intimate partners [7,9,49,56] and concern that others will perceive their vaccination as an indication of ‘promiscuous’ behavior [9,52,55]. In the present study, participants who believed that most people would encourage them to receive an HIV vaccine and who reported they would be motivated to comply with those recommendations were significantly more likely to report MVA. Interestingly, additional research in this sample has indicated that the overwhelming majority of respondents (94%) would be willing to encourage someone to get vaccinated, particularly in circumstances in which the partner was perceived to be at risk or pose a risk for HIV [57]. This finding may serve as preliminary evidence that peer-promotion of HIV vaccination could be a successful strategy for reaching those most at risk for HIV in this population. In this context, the lack of a multivariate association between descriptive norms and vaccine acceptability deserves comment. These data suggest that passive diffusion of vaccine uptake through the social network (i.e. via imitation of others’ behavior) is unlikely, and underscore the importance of an active approach to peer-based promotion.

The findings from this study have several theoretical and methodological implications. This study demonstrates the importance of assessing both the injunctive and descriptive dimensions of social norms and of coupling measures of normative beliefs with assessments of individuals' motivation to comply. Most participants reported that other people would accept an HIV vaccine, but far fewer reported that they would be influenced by others' behavior. Although individuals may underestimate their susceptibility to peer influence, data on compliance with norms may provide preliminary insight into who may be most responsive to strategies such as social marketing.

The research focused on *intent* to receive an HIV vaccine and, until an HIV vaccine is approved, the correspondence between intentions and *actual* vaccine uptake remains unknown. Furthermore, there are limitations of querying respondents about the specific characteristics of a hypothetical vaccine; research employing conjoint analysis [5,6,49,51,58,59] and discrete choice experiments [60] may yield better insight into relative valuations of various vaccine characteristics, project their impact on future acceptability, and inform targeted social marketing campaigns. Though the survey included assessment of several relevant vaccine-related characteristics (e.g., route of administration, dosing, vaccine-induced seropositivity) and included an open-ended item that allowed free-listing of additional barriers to vaccination, the survey did not include direct measures of two vaccine characteristics determined in previous research to be important to acceptability: duration of protection and side effects. Also, while one item measures of theoretical constructs can be problematic to establishing psychometric validity and reliability, the use of scales was not feasible given respondent burden and time constraints for conducting interviews. Similarly, time constraints limited our ability to assess psychosocial correlates to vaccine acceptability by varying levels of vaccine efficacy. The efficacy of future HIV vaccines is currently unknown; however, it is important to specify an efficacy level in measures of vaccine acceptability in order to standardize the context of participants' responses. In the current study, a 90% efficacy level was chosen as it presented a near ‘best case scenario’ for evaluating acceptability given that the ‘most realistic scenario’ is difficult to determine at this stage of vaccine development and subject to change. Nevertheless, more research is needed to explore the generalizability of the findings to vaccines of lower efficacy. Similarly, generalization of findings from this study to other regions of Appalachia and other rural areas in the US also should be made with caution, as sociocultural influences across settings are likely to vary.

## Conclusion

In this rural community, despite low perceived vulnerability to HIV, most drug users were readily willing to accept an HIV vaccine. Minimization of out-of-pocket costs will be essential. Social norms could play a major role in influencing HIV vaccine uptake in this community, and leveraged appropriately, could present an effective mechanism for promoting the vaccine. To plan for effective promotion and dissemination strategies among populations at high risk for HIV, continued research is needed to explore influences on HIV vaccine acceptability among people who use drugs.

## Competing interests

The authors declare that they have no competing interests.

## Authors’ contributions

AMY and JRH designed the study and wrote the protocol. AMY conducted the statistical analyses and drafted the complete manuscript. DSH, RJD, and CES provided feedback throughout the conduct of the study and assisted with editing the final manuscript. All authors contributed to and approved the final manuscript.

## Pre-publication history

The pre-publication history for this paper can be accessed here:

http://www.biomedcentral.com/1471-2458/14/537/prepub
